# Status and predictors of parental food literacy: an Egyptian insight to highlight gaps and challenges

**DOI:** 10.1186/s12889-026-26249-z

**Published:** 2026-02-02

**Authors:** Iman H. Kamel, Ammal M. Metwally, Raefa Refaat Alam, Amani E. Ali, Ghada A. Elshaarawy, Safaa I. Abd El Hady, Abdelrahman K. Hassanein

**Affiliations:** 1 Child Health Department, Medical Research and Clinical Studies Institute, National Research Centre, Dokki, Cairo, Egypt; 2 Community Medicine Research Department, Medical Research and Clinical Studies Institute, National Research Centre, Dokki, Cairo, Egypt; 3 Faculty of Nursing, Mansoura National University, Al Dakhlyia, Egypt; 4 Technical Institute of Nursing, Sherbin, Al Dakhlyia, Egypt; 5 Department of Psychiatry, Ministry of Health and Population, Mansoura General Hospital, El Dakahlyia, Egypt

**Keywords:** Food literacy, Parents, Health literacy, Socioeconomic factors, Body mass index, Feeding behavior, Chronic disease, Public health

## Abstract

**Background:**

There is growing consensus on the importance of food literacy (FL) in public health policies and interventions globally. This study assessed parental total FL status and detected gaps. Determinants contributing to FL adequacy were identified from the socio-demographic, nutrition, and health-related characteristics.

**Methods:**

A cross-sectional study on a diverse cohort of 1000 parents (718 mothers and 282 fathers) using a validated Short FL Questionnaire (SFLQ) was conducted to assess parental total FL status. SFLQ has three sections on functional (FFL; 6 indicators), interactive (IFL; 2 indicators), and critical (CFL; 4 indicators) FL. Parents were selected using multistage stratified random sampling from four governorates representing Egypt’s diverse geographic and socioeconomic contexts. BMI was calculated using self-reported height and weight. The chi-square test, t-test, one-way ANOVA, and logistic regression model were applied to predict parental food literacy.

**Results:**

Participants were 71.8% mothers and 28.2% fathers, with mean ages of 42.2 ± 6.3 and 47.5 ± 5.4 years, respectively. Overall, 58.6% of parents demonstrated adequate TFL, with mothers exhibiting insignificantly a higher adequacy proportion than fathers (60.0% vs. 55.0%, *p* = 0.15). Female parents consistently outperformed male parents across all FL dimensions. However, only 24.5% of parents achieved sufficient awareness levels for total FL, with the lowest proportion observed in the interactive FL domain (18.6%). More than 60% of parents identified indicators related to interactive FL as the most challenging. Multivariate logistic regression analysis identified five significant predictors of adequate FL: regular mineral intake (AOR = 3.97; 95%CI: 2.49–6.33), university-level education or higher (AOR = 2.67; 95%CI: 1.82–3.91), overweight or obesity status (AOR = 1.62; 95%CI: 1.19–2.21), age ≥ 40 years (AOR = 1.59; 95%CI: 1.09–2.32), and higher household crowding index (AOR = 1.52; 95%CI: 1.06–2.17).

**Conclusion:**

The study identified FL status and levels, detecting parental FL challenges in Egypt to be targeted. The study indicated the need for indicators related to IFL and some of FFL to be improved through media-based interventions. Motivating parents to improve their diet quality will help enhance FL to address malnutrition and food insecurity for a more sustainable future for the Egyptian population.

**Supplementary Information:**

The online version contains supplementary material available at 10.1186/s12889-026-26249-z.

## Introduction

The combined challenges of climate change, pollution, biodiversity loss, and the COVID-19 pandemic exacerbated by recent global crises such as Russia’s invasion of Ukraine have created a lasting cost-of-living emergency, increasing food insecurity and food prices worldwide [1]. These converging crises have not only disrupted global food systems but also heightened the urgency of improving population resilience through enhanced nutrition knowledge and behaviours [2, 3]. In this context, food literacy (FL) has emerged as a crucial determinant of individuals’ ability to make informed, sustainable, and health-promoting dietary choices, underscoring its growing recognition as a public-health priority for promoting healthier and more sustainable diets. International organizations such as the World Health Organization (WHO) and the Food and Agriculture Organization (FAO) have emphasized the need to strengthen FL to combat rising diet-related diseases and improve population dietary behaviours [4–6].

FL encompasses the knowledge, skills, and behaviours necessary to make informed food choices, plan and manage meals, and consume foods that meet individual dietary needs while supporting overall health [7–9]. Studies have linked FL to food habits, consumption patterns, and even school performance [10].

Despite global progress, malnutrition remains widespread in low and middle-income countries, including those of the Middle East and North Africa (MENA) region [11]. In Egypt, national data highlight a dual burden of under- and over-nutrition, with 7.8% of children aged 6–12 years being stunted and 8.0% overweight. Maternal education emerged as the most influential determinant for both outcomes [12, 13].

In many low- and middle-income contexts, FL remains an emerging concept [14, 15]. The scarcity of regional data underscores the urgent need to evaluate FL across diverse demographic groups to inform culturally appropriate interventions. Because parents play a central role in shaping children’s dietary behaviours, assessing their FL levels offers an actionable pathway for designing targeted strategies to improve family nutrition and reduce childhood stunting and obesity [4, 5].

While maternal influences on child feeding are well established, the paternal role in nutrition decision-making has been relatively under-explored in Egypt and similar settings. Fathers increasingly contribute to household food purchasing, budgeting, and role-modelling of eating behaviours [16, 17]. Understanding their FL is therefore essential for developing family-centred and gender-responsive interventions. Focusing on paternal FL in this study allows identification of specific gaps, behavioural patterns, and opportunities to engage fathers as equal partners in promoting household nutrition and child health.

Despite growing global recognition of FL’s importance, comprehensive assessments of parental FL in Egypt and the broader MENA region remain scarce. Most existing studies have focused on individual nutrition knowledge or maternal literacy in isolation, without examining the multidimensional nature of FL across diverse parental populations or exploring gender-specific patterns and socioeconomic determinants simultaneously. Furthermore, no previous research has systematically mapped domain-specific gaps in functional, interactive, and critical FL components among Egyptian parents, nor identified the specific indicators that pose the greatest challenges to parental nutrition competency.

Our research question was whether the prevalence of adequate TFL among Egyptian parents differs by sex, what domain-specific gaps exist across functional, interactive, and critical FL, and which socio-demographic, nutritional, and health-related factors predict adequate TFL.

We hypothesized that TFL adequacy is higher among mothers than fathers and is positively associated with higher educational attainment and healthier diet-related behaviors.

Accordingly, the present study aimed to assess parental TFL status and identify domain-specific gaps across its three core components; functional (FFL, six indicators), interactive (IFL, two indicators), and critical (CFL, four indicators) and to examine the socio-demographic, nutritional, and health-related determinants of adequate TFL among Egyptian parents; additionally, the study sought to generate evidence-based recommendations for targeted, culturally appropriate interventions to strengthen parental FL and improve family nutrition outcomes.

This study represents a large multi-governorate, community-based investigation of parental FL in Egypt, offering novel insights into gender differences, socioeconomic gradients, and the relationship between health behaviors and FL competencies, thereby filling a critical knowledge gap and establishing a foundation for evidence-informed public health policy.

## Methodology

### Study design and participants

A cross-sectional study was conducted on a diverse cohort of Egyptian parents to assess their FL. The sample was collected over one year, from Jan 2022 to December 2022. Eligible participants were one of the parents (mother or father) of adolescents aged 10–19 years who were present at home during the interview.

Participants were enrolled from households in the main districts of four governorates representing different geographic regions of Egypt: Cairo (Greater Cairo region), Fayoum (Upper Egypt), Al Dakahleya, Delta region), and Marsa Matrouh (Border/Frontier region). Each region has distinct dietary patterns and cultural habits. Parents were randomly enrolled during a community house-to-house survey. The household sampling frame was derived from a concurrent national screening program for children at risk of autism [18], which provided comprehensive household registries with adolescents aged 10–19 years across multiple governorates (i.e., household lists for field visits in the covered communities, not restricted to screen-positive cases). Importantly, the autism screening served only as a mechanism for household identification and did not influence parent selection or participation in the current study. All parents meeting the inclusion criteria (having at least one adolescent aged 10–19 years and being present at home during the visit) were eligible regardless of their child’s autism screening results. This approach helped ensure that the FL assessment remained independent and broadly reflective of parents in the surveyed communities rather than being restricted to families with particular health concerns.

Details of the selected governorates, localities, and socioeconomic classifications are presented in Supplementary Table S1 (Targeted Households for Parents).

### Sample size and type

A sample size of 895 was calculated [19] to produce a two-sided 98% confidence interval with a distance from the mean (2%) to the limits equal to 0.700 when the estimated standard deviation is 9.0 of Egyptian parental literacy as per the finding of an Arab study [6]. with an added 15% expected loss of indicators to be answered, the sample size was 1030 targeted parents of either a mother or a father. The study sample consisted of 1,000 parents (718 mothers and 282 fathers) with the age range (25–60 and 26–65 years respectively) who completed their questionnaire. The participation rate was 95.24%, with a 4.76% declining rate of participation. 50 participants were enrolled from each social class in the targeted governorate with a total of 150 mothers or fathers per Cairo governorate and 300 per the other 3 targeted governorates. Only one of the two parents from the same house was enrolled in the study.

A multistage stratified random-sampling design was applied to ensure national representativeness. The first stage involved stratifying the sampling frame by socioeconomic status (wealth index) based on the Economic Research Forum and CAPMS classification [20], identifying three strata: low, medium, and high. Within each socioeconomic stratum, cities (Kism) in urban areas and local village units (Markaz) in rural areas were listed. From each stratum, three urban cities and three rural villages were randomly selected using a computer-generated random-number sequence.

In the second stage, within each selected locality, systematic random sampling was employed. Lists of households with adolescents aged 10–19 years were obtained from local health units and community registers in collaboration with the Ministry of Health and Population. From these lists, every household was selected using a predefined sampling interval calculated according to the total number of eligible households in each locality. For each locality, the sampling interval (k) was computed as the ratio of the total number of eligible households to the target number of households to be visited, and the locality-specific values of HH, target sample size, and k are presented in Supplementary Table S1 (Targeted Households for Parents). Within each selected household, one parent; either the mother or the father, depending on who was available was interviewed, ensuring that only one parent per household participated to maintain the independence of observations. This multistage systematic random-sampling approach ensured adequate representation of Egypt’s cultural, dietary, and socioeconomic diversity and enhanced the reproducibility of the study design.

### Study tools and data collection

Data were collected through structured interviews or self-administered questionnaire. The questionnaire was designed to gather information on demographic characteristics, socioeconomic status, FL, vitamin and mineral intake, and health status. The questionnaire was administered in an average of 20 min. The questionnaire was filled in either a printed or an electronic form. Trained field workers conducted the interviews to ensure consistency and accuracy in data collection. The questionnaire was filled out online using survey monkey platform according to the following references:

#### Socio-demographic variables

according to Assad & Kraft, 2012: Data collected on age, gender, education level, marital status, number of children, employment status, monthly income, and household crowding index [21–23]. The household crowding index was calculated as the number of co-residents (excluding newborns) divided by the number of rooms (excluding kitchens and bathrooms).

#### Health status

Chronic diseases were assessed through self-reported data. Participants were asked if they had been diagnosed with any chronic conditions with special emphasis on diabetes, hypertension, or cardiovascular diseases. Vitamin and mineral intake were assessed through self-reported binary questions regarding regular use of dietary supplements. Participants were asked whether they regularly consumed vitamin supplements (including vitamins A, C, D, B12, and folic acid) and mineral supplements (including calcium, iron, magnesium, and zinc) with yes/no response options. This assessment reflected supplement use only and did not quantify daily intake amounts, evaluate dietary sources, or assess adequacy against recommended dietary allowances.

#### FL

Parental FL was assessed using a validated FL questionnaire, which included sections on functional, interactive, and critical FL. The Arabic version of the short FL questionnaire (SFLQ) according to Gréa Krause et al. 2020 and Koca and Arkan 2020 were used for parental FL (FL) assessment [24, 25], The Arabic version of the questionnaire that was adapted to the local context was used before by Hoteit and her colleagues [6] in a multi-country Arab population sample that included Lebanese, Jordanian, and other MENA populations. However, it had not been specifically validated among Egyptian parents prior to this study. Therefore, we re-tested internal consistency in our sample. The SFLQ demonstrated good internal consistency with Cronbach’s alpha values of 0.79 (total scale), 0.74 (functional), 0.71 (interactive), and 0.76 (critical). These findings support the scale’s internal consistency and usability in the present Egyptian parent population. In addition, the questionnaire was reviewed by three Egyptian nutrition experts and piloted with 30 parents (excluded from the final analysis) to ensure cultural appropriateness and comprehension. We also added a limitation noting that future work should conduct comprehensive psychometric validation in Egypt (e.g., construct validity and test–retest reliability).

FL questionnaire was used to evaluate the parents’ ability to follow national and international sources regarding food information and apply this information in their daily lives to promote healthier eating behaviors. It includes 12 indicators with three FL dimensions: functional FL (FFL; 6 indicators), interactive FL (IFL; 2 indicators), and critical FL (CFL; 4 indicators). Each question has a score range of 4- or 5-point Likert scale: strongly disagree to strongly agree and a higher score indicates better FL. The scoring criteria of the parental FL range from 7 to 52 with an average score ≥ 36. Moreover, the FFL sub-dimension assesses the parents’ food practical skills and knowledge. The IFL sub-dimension explores the parents’ ability to participate in discussions and make decisions about food choices for their entire family. While CFL sub-dimension focuses on parents’ critical thinking skills related to food and nutrition. Scores were calculated for each section and an overall FL score was derived.

#### The indicators under each component of parental FL are as follows

Six indicators (5 awareness and one practice) under the FFL include: Knowing where one can get the information needed when having indicators or concerns about proper nutrition; Understanding nutritional information presented in the form of leaflets (e.g., labels, brochures), nutritional facts labeled on food products, nutrition programs on television or radio, dietary recommendations from nutrition professionals, or nutritional advice from family members or friends; Knowing the food pyramid well; Awareness of the official recommendations regarding fruit and vegetable consumption; Awareness of the official recommendations regarding salt consumption in food; Difficulty of preparing a complete meal with nutrients (such as proteins, starches, healthy fats, and vegetables).

#### The IFL includes two indicators

The ability to help a family member or friend with indicators about nutrition; and the ability to select the information about proper nutrition that seems right and relevant.

The CFL includes four indicators: Difficulty in evaluating the reliability of nutritional information found on websites or transmitted through the media (internet, social media, television, radio…); Difficulty in evaluating the validity of the advertisements that may link certain food products to how they affect health; Difficulty in determining whether a certain food fits with a healthy diet; Difficulty in evaluating how your current eating choices and habits affect your long-term health.

#### Body Mass Index (BMI)

BMI was calculated using self-reported height and weight. Participants were categorized into underweight, normal weight, and overweight/obese based on the World Health Organization (WHO) BMI classification. The BMI was calculated as weight (in kilograms) divided by height (in meters) squared based on the WHO growth standards with the help of the Anthro-Program of PC [26]. The classification of Parents’ BMI was as follows: underweight if BMI is less than 18.5, normal/healthy weight if BMI is 18.5 to 24.9, overweight if BMI is 25.0 to 29.9, and obese if BMI is 30.0 or higher [27].

### Data processing

The studied participants were classified into four categories according to their acquired score in each component of FL: ≤25%, > 25%- 50%, > 50%- 75%, and > 75%. The total food-literacy score was calculated by summing responses across all 12 indicators of the SFLQ, with possible scores ranging from 7 to 52 points. Based on the validated scoring criteria for the Arabic version of the SFLQ, parents were categorized into two outcome groups: adequate food literacy (FL) (total score ≥ 36) and poor FL (total score < 36). This cut-off enables a clear distinction between parents who achieve a satisfactory level of FL and those requiring targeted interventions. The awareness level of each indicator will determine the objectives to be achieved to increase awareness of the FL set of indicators. When 50% or more participants were unaware of one or more Indicators, this set of indicators is a gap and challenging to achieve. According to the assessment results, the target objectives were set to be realistic and achievable: For any indicator whose measure was 50% or less, the target has to reach 75%. For any indicator measuring > 50% to 75%, the target will be set to reach > 70–80%. For any indicator measuring > 75%, the target is to sustain the indicator. The total FL score was calculated by summing responses across all 12 indicators of the SFLQ, with possible scores ranging from 7 to 52 points. Based on the validated scoring criteria for the Arabic version of the SFLQ, parents were categorized into two groups [6]: adequate FL (total score ≥ 36) and poor FL (total score < 36). This dichotomization allows for clear identification of parents who have achieved a satisfactory level of FL versus those who require targeted interventions.

### Statistical analysis

Data were analyzed using Statistical Package for the Social Sciences (SPSS) version 26.0 [28]. Frequency and percentages were computed for comparisons between qualitative variables. Continuous data was expressed as mean and standard deviation (SD). Pearson’s chi-square test (χ²), Fisher’s exact test, and Z test were used for qualitative comparisons, while independent *t*-tests and one-way ANOVA were used to compare mean values across groups.

To evaluate the degree of association between parental BMI, vitamin and mineral intake, and FL, odds ratio (OR) with 95% confidence interval (CI) were calculated. These variables were examined bidirectionally; both as correlates and predictors to explore their associative relationships with FL rather than implying causation. Multivariable logistic regression analysis was performed to identify the significant predictors of adequate total parental FL among the studied parents. Missing values were handled using complete-case (listwise) deletion within each analysis, and denominators are presented as available. For multivariable modelling, predictors with zero-cell counts or no meaningful variability (i.e., complete or quasi-complete separation) were not entered into the regression model; accordingly, job status was excluded because all fathers were employed, making this variable non-informative and highly collinear with gender. Potential multicollinearity among predictors was assessed using variance inflation factor (VIF) and tolerance values. All variables demonstrated acceptable collinearity thresholds (VIF < 2.5; tolerance > 0.4), confirming the absence of significant multicollinearity. A *p*-value < 0.05 was considered statistically significant.

## Results

### Demographic characteristics, socioeconomic factors, health status, and nutritional supplementation

Of the targeted eligible sample, 1,000 parents completed the interview (participation rate: 95.24%), while 50 parents declined participation (4.76%). Table 1 presents the characteristics of the 1,000 participating parents, stratified by gender and organized into three thematic domains.

**Table 1:** Parental socio-demographic, health, and nutrition status characteristics according to gender

Variables	Mothers(*n*=718; 71.8%)*n* (%)	Fathers(*n*=282; 28.2%)*n* (%)	*P* value
Age by years (mean ± SD)	42.2 ± 6.3	47.5 ± 5.4	<0.001**
Marital status			
Married	611 (85.1)	275 (97.5)	<0.001**
Not married (Divorced or Widowed)	107 (14.9)	7 (2.5)	
Number of children			
≤ 3 children	638 (88.9)	260 (92.2)	0.116
> 3 children	80 (11.1)	22 (7.8)	
Household crowding index^§^			
Not crowded (≤ 1)	513 (71.4)	76 (27.0)	<0.001**
Crowded (> 1)	205 (28.6)	206 (73.0)	
Education level			
Never attend school	9 (1.3)	29 (10.3)	<0.001**
School level	114 (15.9)	97 (34.4)	
University level	595 (82.9)	156 (55.3)	
Job-status			
Not employed	50 (7.0)	0 (0.0)	<0.001**
Employed (Full-time job, Part-time job,	668 (93.0)	282 (100.0)	
Self-employed)			
Monthly income			
Less than 5000 EP	100 (35.5)	172 (61.0)	<0.001**
5000- 10000 EP	72 (25.5)	55 (19.5)	
More than 10000 EP	110 (39.0)	55 (19.5)	
History of chronic diseases			
No	226 (31.5)	147 (52.1)	<0.001**
Yes	492 (68.5)	135 (47.9)	
Weight status (BMI)			
Underweight	13 (1.8)	1 (0.4)	0.038*
Normal-weight	206 (28.7)	99 (35.1)	
Overweight / obese	499 (69.5)	182 (64.5)	
Vitamin Intake			
No	489 (68.1)	139 (49.3)	<0.001**
Yes	229 (31.9)	143 (50.7)	
Mineral Intake			
No	551 (76.7)	236 (83.7)	0.016*
Yes	167 (23.3)	46 (16.3)	

§Household crowding index = number of co-residents (excluding newborns) ÷ number of rooms (excluding kitchens and bathrooms) [22, 23]. Test of significance: χ² test, Fisher’s exact test, and t-test between two means

*Significance levels: *p* < 0.05 (significant); **p < 0.01

#### Sociodemographic profile

Most parents were aged ≥ 40 years (59.1%), with mothers representing 61.8% of participants. Most mothers were employed (93.0%), while all fathers were employed (100.0%); only 7.0% of mothers reported not being employed (*p* < 0.001). University education was reported by 82.9% of mothers and 55.3% of fathers (*p* < 0.001). The majority of families belonged to the medium socioeconomic category (56.3%) and lived in non-crowded households.

#### Health-related characteristics

Chronic diseases were significantly more prevalent among mothers (68.5%) than fathers (47.9%, *p* < 0.001). Overweight and obesity were common in both groups, affecting 69.5% of mothers and 64.5% of fathers (*p* = 0.038).

#### Nutritional supplementation

Nutritional Supplementation: Fathers reported higher vitamin supplement use than mothers (50.7% vs. 31.9%, *p* < 0.001), while mineral supplement intake was higher among mothers than fathers (23.3% vs. 16.3%, *p* = 0.016).

**Fig. 1:**
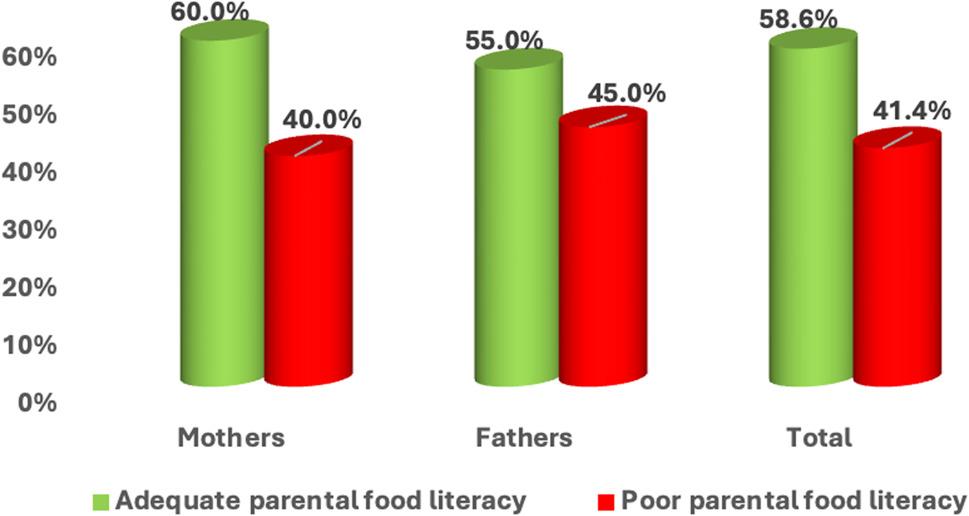
Percent distribution of parental total FL (SFLQ score categories: adequate ≥ 36; poor < 36) according to gender. Test of significance: Z test between two proportions comparing the prevalence of adequate FL between mothers and fathers (*p* = 0.15)

Figure 1 shows the distribution of parental food literacy (FL) by gender based on the total Short Food Literacy Questionnaire (SFLQ) classification (adequate FL ≥ 36; poor FL < 36). Overall, 58.6% of parents demonstrated adequate FL and 41.4% had poor FL (descriptive). Adequate FL was 60% among mothers and 55% among fathers. The difference in the prevalence of adequate FL between mothers and fathers was assessed using a Z test for two proportions (*p* = 0.15).

### Mean scores and awareness levels of FL components

Female parents consistently scored significantly higher across all FL components (all *p* < 0.001): FFL (17.6 ± 3.7 vs. 15.9 ± 4.3), IFL (6.6 ± 2.4 vs. 5.8 ± 2.1), CFL (12.0 ± 2.5 vs. 10.4 ± 3.1), and total FL (36.2 ± 7.8 vs. 32.1 ± 9.0). Overall, only 24.5% of parents achieved sufficient awareness for total FL. Component-specific analysis revealed that awareness remained limited for FFL (20.4% sufficient), IFL (18.6% sufficient), and CFL (20.3% sufficient). Examination of individual indicators highlighted that more than half of the parents exhibited poor awareness in several domains (Table 2). Within the IFL component, 62.4% were unaware of how to select appropriate nutrition information, and 68.3% lacked the ability to help family or friends with such information, indicating weak communication and information-sharing skills. For FFL, three of six indicators were identified as challenging: understanding nutritional facts on food labels (68.8% unaware), knowing where to obtain reliable nutrition information (61.2% unaware), and knowledge of the food pyramid (56.1% unaware). By contrast, CFL showed comparatively better performance, with most indicators falling in the neutral range (25–50% unaware) and one indicator in the sufficient range (< 25% unaware), reflecting relatively stronger evaluation and judgment skills. Collectively, these results underscore that parents possess higher critical reasoning than functional or interactive competencies, emphasizing the need for interventions that strengthen practical nutrition comprehension and application within households.

**Table 2:** Awareness indicators level of components of parental FL

Determinants#	Parental total FL(*n*=1000) *n* (%)
Challenging category (> 50% were unaware):	
FFL component include:	
1. Knowing where one can get the information needed when having concerns about proper nutrition.	612 (61.2)
2. Understanding nutritional information and facts presented in leaflets, on television or radio, from nutrition professionals, or from family members or friends.	688 (68.8)
3. Knowing the food pyramid well.	561 (56.1)
IFL sub-dimension includes:	
4. The ability to help a family member or friend with information about nutrition.	683 (68.3)
5. The ability to select the information about proper nutrition that seems right and relevant.	624 (62.4)
Neutral category (25-50% were unaware or inexperienced):	
FFL sub-dimension includes:	
6. Awareness of the official recommendations regarding fruit and vegetable consumption	345 (34.5)
7. Awareness of the official recommendations regarding salt consumption in food	357 (35.7)
8. Difficulty of preparing a complete meal with nutrients (such as proteins, starches, healthy fats, and vegetables	330 (33.0)
CFL sub-dimension includes:	
9. Difficulty in evaluating the reliability of nutritional information found on websites or transmitted through the media (internet, social media, television, radio...	310 (31.0)
10. Difficulty in evaluating the validity of the advertisements that may link certain food products to how they affect health.	275 (27.5)
11. Difficulty in determining whether a certain food fits with a healthy diet;	285 (28.5)
Sufficient category (<25% were unaware or inexperienced):	
CFL sub-dimension includes:	
12. Difficulty in evaluating how your current eating choices and habits affect your long-term health	245 (24.5)

# Determinants refers to the individual FL indicators/items assessed within each domain (FFL/IFL/CFL) and categorized by the proportion of parents reporting being unaware/inexperienced (challenging/neutral/sufficient)

### Correlates of FL components

As shown in Table 3, age, gender, family structure, socioeconomic status, and nutritional supplementation showed significant associations with multiple FL domains. Parents aged ≥ 40 years had higher CFL scores (11.7 ± 2.8 vs. 11.1 ± 2.6, *p* = 0.011), while female parents consistently scored higher across all components (all *p* < 0.001). Larger family size (> 3 children) and lower crowding indices were associated with better functional and interactive FL (*p* < 0.05 and *p* < 0.001, respectively).

Educational attainment and income level demonstrated strong positive gradients—university-educated and higher-income parents achieved significantly higher FL scores across all components (all *p* < 0.001).

Unemployed parents also exhibited higher FL scores than employed parents (all *p* < 0.001), suggesting greater availability for food-related tasks.

Although chronic disease presence did not significantly influence FL, overweight and obese parents displayed higher scores across components (all *p* < 0.05).

Regular vitamin and mineral supplement use was strongly associated with improved FL in all domains (all *p* < 0.001). These findings collectively highlight the influence of socioeconomic and lifestyle factors on parental FL, underscoring the role of education, income, and household environment in shaping FL outcomes.

**Table 3:** Correlates of the epidemiological factors and supplement intake with parental FL components

Variables	FFL^(a)^(*n*=1000)(mean ± SD)	IFL^(b)^(*n*=1000)(mean ± SD)	CFL^(c)^(*n*=1000)(mean ± SD)	Total FL^(d)^(*n*=1000)(mean ± SD)
Age group				
< 40 years	16.9 ± 3.6	6.1 ± 2.1	11.1 ± 2.6	34.1 ±7.9
≥ 40 years	17.2 ± 4.0	6.4 ± 2.4	11.7 ± 2.8	35.3 ± 8.4
*P* value	0.364	0.161	0.011*	0.094
Gender				
Female	17.6 ± 3.7	6.6 ± 2.4	12.0 ± 2.5	36.2 ± 7.8
Male	15.9 ± 4.3	5.8 ± 2.1	10.4 ± 3.1	32.1 ± 9.0
*P* value	<0.001**	<0.001**	<0.001**	<0.001**
Marital status				
Married	17.1 ± 3.9	6.4 ± 2.3	11.6 ± 2.8	35.1 ± 8.2
Not married (Divorced or Widowed	17.1 ± 4.4	6.2 ± 2.6	11.4 ± 3.0	34.7 ± 9.2
*P* value	0.835	0.498	0.458	0.590
Number of children in the household				
≤ 3 children	17.1 ± 3.9	6.3 ± 2.4	11.5 ± 2.8	34.9 ± 8.5
> 3 children	17.9 ± 3.8	7.0 ± 1.7	11.9 ± 2.1	36.8 ± 6.5
*P* value	0.043*	0.001**	0.053	0.026*
Household crowding index^§^				
Not crowded (≤ 1)	17.6 ± 3.9	6.5 ± 2.6	12.0 ± 2.7	36.1 ± 8.3
Crowded (> 1)	16.5 ± 4.0	6.2 ± 2.0	10.9 ± 2.8	33.6 ± 8.2
*P* value	<0.001**	0.061	<0.001**	<0.001**
Education level				
School level	15.5 ± 3.5	5.6 ± 2.0	10.4 ± 2.7	31.5 ± 7.5
University level or higher	17.9 ± 3.6	6.7 ± 2.4	12.2 ± 2.4	36.8 ± 7.5
*P* value	<0.001**	<0.001**	<0.001**	<0.001**
Job status				
Not employed	20.5 ± 4.4	7.3 ± 2.2	13.1 ± 2.5	40.8 ± 8.3
Employed (Full-time job, Part-time job, Self-employed)	17.0 ± 3.8	6.3 ± 2.4	11.5 ± 2.8	34.8 ± 8.2
*P* value	<0.001**	0.004**	<0.001**	<0.001**
Monthly income				
Less than 5000 EP	15.5 ± 4.4	5.5 ± 2.4	10.1 ± 3.4	31.1 ± 9.5
More than 5000 EP	17.5 ± 3.7	6.6 ± 2.3	11.9 ± 2.5	36.0 ± 7.7
*P* value	<0.001**	<0.001**	<0.001**	<0.001**
Parent has one or more chronic disease.				
No	17.2 ± 4.0	6.4 ± 2.0	11.4 ± 2.6	35.0 ± 7.9
Yes	17.1 ± 3.9	6.3 ± 2.5	11.7 ± 2.9	35.1 ± 8.6
*P* value	0.565	0.478	0.108	0.948
Weight status (BMI)				
UnderweightNormal weight	16.1 ± 2.316.7 ± 4.8	5.7 ± 0.66.0 ± 2.4	11.1 ± 1.610.8 ± 3.3	33.0 ± 3.733.4 ± 10.0
Overweight/obese	17.4 ± 3.5	6.5 ± 2.4	11.9 ± 2.5	35.8 ± 7.4
*P* value	0.025*	0.001**	<0.001**	<0.001**
Vitamin Intake				
No	16.6 ± 4.2	5.9 ± 2.5	11.1 ± 2.9	33.6 ± 8.8
Yes	18.1 ± 3.3	7.1 ± 2.0	12.4 ± 2.4	37.6 ± 6.7
*P* value	<0.001**	<0.001**	<0.001**	<0.001**
Mineral Intake				
No	16.6 ± 3.9	6.0 ± 2.3	11.2 ± 2.7	33.8 ± 8.2
Yes	19.0 ± 3.3	7.7 ± 2.2	13.1 ± 2.6	39.7 ± 7.0
*P* value	<0.001**	<0.001**	<0.001**	<0.001**

§Household crowding index = number of co-residents (excluding newborns) ÷ number of rooms (excluding kitchens and bathrooms) [22, 23]. Test of significance: t-test between two means and ANOVA between groups. *Significance levels: * *p* < 0.05; **p < 0.01), (a) Functional FL; (b) Interactive FL; (c) Critical FL; (d) Total FL

### Associations between parental FL and health-related factors

As shown in Table 4, parents with poor FL were significantly more likely to be underweight (OR = 3.3, 95%CI: 0.9–12.0), whereas those with adequate FL were more frequently overweight or obese (OR = 0.5, 95%CI: 0.4–0.6, *p* < 0.01). Poor FL was also associated with lower supplement use: non-use of mineral supplements (OR = 3.8, 95%CI: 2.6–5.5, *p* < 0.01) and vitamin supplements (OR = 2.4, 95%CI: 1.5–3.2, *p* < 0.01).

**Table 4:** Bivariate associations between parental TFL and selected health-related variables

Variables	Studied parents (n=1000)						
	BMI			Vitamins intake		Minerals intake	
	Underweight *n*= 14, *n* (%)	Overweight/ Obese *n*= 681, *n* (%)	Normal weight *n*= 305, *n* (%)	No *n*=628 *n* (%)	Yes *n*=372 *n* (%)	No *n*=787 *n* (%)	Yes *n*=213 *n* (%)
Parental FL							
Poor (*n*=414)	11 (78.6)	242 (35.5)	161 (52.8)	308 (49.0)	106 (28.5)	373 (47.4)	41 (19.2)
Adequate (*n*=586)	3 (21.4)	439 (64.5)	144 (47.2)	320 (51.0)	266 (71.5)	414 (52.6)	172 (80.8)
OR (95% CI)	3.3 (0.9-12.0)	0.5 (0.4-0.6) **		2.4 (1.5-3.2) **		3.8 (2.6-5.5) **	

Test of significance: χ² test and odds ratio (OR) with 95% confidence interval (CI), Significance levels: * *p* < 0.05; **p < 0.01)

### Predictors of adequate FL

Table 5 presents the multivariable logistic regression model (enter method) examining independent predictors of adequate TFL among parents. The model demonstrated a good overall fit (χ² = 98.4, *p* < 0.001; Nagelkerke *R*² = 0.42). Using the table’s stated reference categories (< 40 years, female, married, ≤ 3 children, not crowded (≤ 1), school education, not employed, income < 5000 EGP, normal-weight), four variables remained statistically significant in the adjusted model. Parents aged ≥ 40 years had higher odds of adequate TFL compared with those < 40 (AOR = 1.59, 95%CI: 1.09–2.32, *p* = 0.016). Parents with university education or higher were more likely to demonstrate adequate TFL than those with school education (AOR = 2.67, 95%CI: 1.82–3.92, *p* < 0.001). Living in a crowded household (> 1) was associated with higher odds of adequate TFL compared with not crowded households (AOR = 1.52, 95%CI: 1.06–2.17, *p* = 0.012). Regarding weight status, overweight/obesity was associated with higher odds of adequate TFL versus normal weight (AOR = 1.62, 95%CI: 1.19–2.21, *p* = 0.002), while underweight was not significant (*p* = 0.799). Mineral intake showed a strong positive association with adequate TFL (AOR = 3.97, 95%CI: 2.49–6.33, *p* < 0.001), whereas vitamin intake was not significant (*p* = 0.859). Gender, marital status, number of children,, monthly income, and having ≥ 1 chronic disease were not significant predictors in the multivariable model (all *p* > 0.05).

**Table 5:** Multivariable logistic regression model for prediction of adequate parental TFL

Variables	β	AOR	*P* value	95% CI for AOR	
				Lower	Upper
Age group					
< 40 years (ref.)		1			
≥ 40 years	0.465	1.592	0.016	1.092	2.321
Gender					
Females (ref.)		1			
Males	0.152	1.164	0.434	0.796	1.702
Marital status					
Married (ref.)		1			
Not married (Divorced or Widowed)	0.145	0.156	0.550	0.718	1.860
Number of children in the household					
≤ 3 children (ref.)		1			
> 3 children	0.172	1.188	0.503	0.718	1.968
Household crowding index					
Not crowded (≤ 1) (ref.)		1			
Crowded (> 1)	0.419	1.520	0.012	1.064	2.173
Education level					
School level (ref.)		1			
University level or higher	0.981	2.667	<0.001	1.816	3.916
Monthly income					
Less than 5000 EP (ref.)		1			
More than 5000 EP	0.340	1.405	0.149	0.885	2.231
Parent has one or more chronic disease					
No (ref.)		1			
Yes	-0.103	0.902	0.548	0.645	1.262
Weight status (BMI)					
Normal-weight (ref.)		1			
Underweight	-0210	0.810	0.799	0.161	4.089
Overweight/obese	0.483	1.621	0.002	1.187	2.213
Vitamins intakeNo (ref.)					
No		1			
Yes	-0.034	0.966	0.859	0.661	1.411
Minerals intake					
No (ref.)		1			
Yes	1.379	3.970	<0.001	2.488	6.333
Constant			0.000		

Variable(s) entered in each model: age, gender, BMI, education level, marital status, number of children, job status, monthly income, household crowding index, one or more chronic diseases, vitamin intake, and mineral intake. Test of significance: Binary logistic regression model.AOR Adjusted Odds Ratio,CI Confidence Interval, (ref.) indicates the reference category (AOR = 1.00) as labelled in the table. Significance levels: *p* < 0.05; *p* < 0.01)

## Discussion

This study provides the first comprehensive assessment of parental FL in Egypt, identifying key gaps, strengths, and determinants across a diverse population sample. The analysis revealed clear gender-specific differences, distinctive behavioural patterns in supplement use, and multifactorial relationships between sociodemographic characteristics, health status, and FL adequacy. These insights carry important implications for developing targeted, evidence-based interventions aimed at enhancing nutritional knowledge, decision-making, and healthy behaviours among Egyptian parents.

### FL awareness level

The present study revealed that only 24.5% of parents achieved sufficient awareness of TFL, while more than half demonstrated limited understanding of its functional and interactive dimensions. IFL showed the weakest performance, with over 60% of parents reporting difficulty in selecting appropriate nutrition information or assisting others in interpreting it. This pattern underscores substantial gaps in practical and communication-based nutrition competencies among Egyptian parents.

Comparable findings have been reported in regional studies showing that individuals often possess basic nutritional knowledge but lack the capacity to apply or communicate it effectively [29–32]. In our context, limited access to culturally tailored educational materials and a scarcity of family-cantered nutrition programs may contribute to this gap. In line with previous research from Egypt and other MENA countries [30, 33, 34], the present results highlight that functional literacy (reading labels, identifying nutrient content) remains insufficient, while critical evaluation skills are relatively better developed.

These results have direct implications for intervention design. Public health initiatives should prioritize improving parents’ practical food-handling and interpretation skills through community-based workshops and school-linked programs that emphasize the everyday application of nutrition information [30, 33–35]. Integrating FL modules into school curricula and primary healthcare counselling has been shown to strengthen both functional and interactive competencies and to enhance nutrition-related decision-making at the household level [36–38]. Tailored interventions should also consider gender and socioeconomic differences observed in this population to ensure equitable reach and impact, as earlier studies have underscored the modifying role of these determinants in shaping FL and dietary behaviours [6, 39–42]. A comprehensive scoping review of food and nutrition literacy programs published in 2023 also highlighted the effectiveness of various educational initiatives in improving FL [43].

### FL status

There are some controversial results about gender literacy. Our findings align with previous studies indicating that women generally have higher levels of nutritional knowledge and food literacy compared to men [6, 39–47]. Women were also reported to be better at critically assessing the reliability and validity of nutritional information compared to men [29]. It was found that women were more likely to engage in discussions about nutrition and seek out nutritional information compared to men [46]. However, other studies did not find gender differences in food literacy [42, 48].

However, when adjusting for potential confounders, the strength of these associations shifted. Although female parents demonstrated higher mean scores for functional, interactive, and critical food literacy components in the bivariate analysis, gender did not retain statistical significance as an independent predictor of TFL in the multivariable regression model. This attenuation suggests mediation by socioeconomic variables—particularly education, employment, and household income. Similarly, several other variables that showed significant bivariate associations (e.g., BMI and supplement use) lost significance in the adjusted model, indicating potential confounding by socioeconomic position and access to nutrition information.

The documented gender difference in our study may also reflect traditional gender roles, where women often take primary responsibility for food preparation and nutrition within households. These findings underscore the importance of gender-sensitive educational strategies that empower both parents to strengthen their FL and promote equitable household nutrition decisions.

### Health-related correlates of FL

Both nutritional supplement intake and weight status were strongly related to FL, functioning as both predictors and outcomes. Fathers reported higher vitamin supplement use than mothers (50.7% vs. 31.9%, *p* < 0.001), while mineral supplement intake was also slightly higher among fathers (16.3% vs. 23.3%, *p* = 0.016. These findings align with research demonstrating that supplement use reflects greater nutrition awareness and self-efficacy [49–51].

Interestingly, the study found that both overweight/obese and unemployed parents exhibited higher FL levels than their counterparts, findings that appear counterintuitive at first glance. In the case of overweight and obese parents, this relationship may reflect heightened health awareness and self-monitoring behaviours following exposure to medical advice, dietary counselling, or chronic disease risk awareness. This could enhance FL without necessarily translating into behavioural change or weight loss. Several studies have similarly reported that individuals with diet-related conditions often acquire greater nutrition knowledge through treatment or self-education, yet struggle with sustained dietary adherence or lifestyle modification [6, 50, 51].

Likewise, unemployed parents demonstrated higher FL scores than employed counterparts. This may be explained by increased time availability, greater involvement in meal planning and preparation, or caregiving responsibilities; especially among mothers allowing more opportunities to apply FL skills. Similar observations have been reported in population-based studies where greater time for food-related activities was associated with improved FL, cooking confidence, and dietary diversity [52–54]. Alternatively, this finding could also indicate that employment constraints and time scarcity among working parents reduce opportunities to apply or practice FL skills in daily life, a pattern supported by prior research on time-related barriers to healthy food behaviours [55, 56].

Thus, while unemployment may coincide with higher FL scores, it does not necessarily imply healthier overall lifestyles, emphasising the need for workplace-based nutrition education and time-efficient strategies that enable employed parents to maintain adequate FL and dietary quality. Moreover, higher FL does not always equate to healthier dietary practices or optimal body weight. Instead, they highlight the complex interplay between knowledge, behaviour, and contextual constraints emphasizing the need for integrated interventions that combine FL education with behavioural and environmental supports to facilitate healthier outcomes.

### Socioeconomic determinants of FL

The findings of our study highlight the multifaceted nature of FL and the influence of demographic and socioeconomic factors. Education, income, employment status, and household conditions collectively shaped parental FL levels, confirming similar global trends [57–60]. Parents aged 40 years and above had significantly higher mean scores for CFL compared to those under 40 years, reflecting greater experience and exposure to health information over time [40, 41]. Likewise, parents with a university-level education or higher exhibited substantially higher FL scores across all domains. Education remained the strongest independent predictor in the regression model (AOR = 2.67; 95%CI: 1.82–3.91), reinforcing its critical role in shaping health literacy globally [57–60].

Monthly income and employment status were significantly associated with FL in the bivariate analysis but not as independent predictors after adjustment, suggesting that education mediates their influence. The relationship between household crowding and FL presented an intriguing pattern: while bivariate analyses showed lower FL scores among larger households, regression analysis revealed that moderate crowding predicted higher odds of adequate FL (AOR = 1.52; 95%CI: 1.03–2.24). This reversal likely reflects the confounding effect of socioeconomic status—smaller households in our sample were also more affluent and highly educated. After adjustment, the positive association suggests that shared living environments may foster collective food-related learning, intergenerational knowledge transfer, or improved resource management, all of which can enhance FL [14, 61–63].

The domain-specific patterns observed in this study provide insight into how identified determinants shape different facets of FL. Higher educational attainment was most strongly associated with better interactive and critical food literacy. This supports evidence that education enhances individuals’ capacity to interpret, communicate, and evaluate nutrition information rather than simply recall it [61, 64, 65]. Conversely, parents with lower education may possess basic functional skills yet lack the confidence or communication strategies needed to discuss nutrition effectively with family members. This explains the particularly low IFL scores observed in this group. Moreover, mineral-supplement users scored higher across all domains, possibly reflecting greater exposure to health counselling, medical advice, or self-directed information seeking, which reinforces interactive and critical competencies [52, 66, 67]. These findings indicate that interventions targeting at-risk groups; such as less-educated parents or those with limited healthcare engagement should prioritize interactive skills, including how to locate, discuss, and translate nutritional information into practical household behaviours.

### Implications for public health policy

The findings of this study have significant implications for strengthening public health strategies in Egypt and similar middle-income contexts. Enhancing parental FL requires a multi-level approach that combines education, community engagement, and supportive policy frameworks.

At the program level, interventions should integrate FL training into primary healthcare and school-based programs, expand community learning initiatives, and design gender-responsive modules that empower both mothers and fathers as active nutrition gatekeepers [38, 57, 59, 68–70]. Given that fathers in this study reported higher supplement use but lower FL scores, male-focused awareness and counselling programs could effectively bridge behavioural–knowledge gaps and promote shared responsibility for family nutrition [33, 46, 71–74].

At the policy level, FL should be mainstreamed within national nutrition and health promotion agendas through intersectoral collaboration among the Ministries of Health, Education, and Social Solidarity. Policymakers should ensure equitable access by prioritizing low-income and less-educated families [2, 38, 57, 75] and incorporating FL indicators into national monitoring systems to evaluate progress [36–38].

At the household and community levels, improving parental FL can generate intergenerational benefits—parents with higher FL are more capable of modelling healthy eating, guiding children’s food choices, and fostering dietary diversity [49, 61, 63, 66]. Evidence from Egyptian adolescents also confirms that adequate parental FL substantially increases adolescent nutrition literacy and micronutrient adequacy, underscoring the importance of family-cantered and life-course-oriented interventions [43].

Beyond its individual and household-level importance, parental FL exerts a critical intergenerational influence on children’s nutrition literacy, dietary diversity, and health behaviours. Parents with higher FL demonstrate better capacity to model healthy eating, structure balanced meals, and guide children’s food choices, thereby improving adolescents’ Total Nutrition Literacy and related health outcomes [69]. Evidence from Egyptian adolescents shows that adequate parental FL nearly doubled the odds of achieving adequate TNL and significantly increased vitamin and mineral intake, confirming its pivotal role in shaping youth dietary outcomes. These findings underscore the need for family-cantered nutrition-education policies that empower both mothers and fathers as co-educators and role models in promoting healthy eating patterns across generations.

Strengthening FL across these levels can ultimately contribute to healthier dietary behaviours, improved nutrition outcomes, and the reduction of Egypt’s dual burden of undernutrition and obesity [12, 13, 33, 35, 76].

### Strengths and limitations

This study, while providing valuable insights into the factors influencing parental FL, among Egyptian parents, has some limitations that should be acknowledged: First, its cross-sectional design precludes establishing causal relationships between FL and its associated variables. Accordingly, variables such as BMI, vitamin intake, and mineral intake were analysed as correlates rather than direct outcomes or causes of FL. The observed associations should therefore be interpreted as descriptive and associative, not causal. The use of an autism screening program’s household registry as a sampling frame did not introduce systematic bias, as parent selection was independent of screening outcomes and based solely on the presence of adolescents in the household.

The reliance on self-reported data for assessing FL and dietary supplement intake may introduce bias, potentially leading to overestimation or underestimation of responses.

Although the FL questionnaire was validated and culturally adapted, it may not have captured the full spectrum of FL dimensions. Similarly, vitamin and mineral intake was assessed through simple self-reported supplement use rather than comprehensive dietary tools, limiting the ability to evaluate total nutrient intake and adequacy. Future studies should employ validated dietary assessment instruments, such as food frequency questionnaires or repeated 24-hour dietary recalls, to generate more accurate nutrient profiles. Although the SFLQ demonstrated good reliability in our sample (Cronbach’s α = 0.79), future research should conduct comprehensive validation studies including construct validity and test-retest reliability specifically for the Egyptian context.

Despite the use of a multistage stratified random sampling approach to ensure representation across Egypt’s main regions and socioeconomic strata, participation depended on the availability of one parent during household visits; typically, the parent present at home during daytime hours which may have introduced minor selection bias, particularly underrepresenting employed fathers. Nevertheless, the probabilistic sampling design and large sample size helped mitigate this limitation. Additionally, several potentially confounding variables, such as physical activity levels, detailed dietary patterns, and broader lifestyle behaviours, were not assessed and may have influenced the observed associations. Future longitudinal studies incorporating these variables could better clarify causal pathways and contextual determinants of FL among Egyptian parents.

Despite these limitations, the study has several notable strengths. It is the first nationally representative investigation of parental FL in Egypt, encompassing diverse geographic, cultural, and socioeconomic contexts. The multistage stratified design enhanced representativeness and generalizability of the findings across Egypt’s population.

This study also provides the first empirical mapping of specific gaps in parental FL indicators; particularly functional and interactive competencies thereby offering actionable insights for targeted public health interventions. Furthermore, the large sample size and comprehensive analytic approach, including multivariable regression modeling, enabled robust identification of key predictors of FL.

Key determinants identified; such as age, educational attainment, mineral supplement intake, and overweight/obesity highlight the multidimensional nature of FL and its links to both sociodemographic and health-related factors. Collectively, these insights establish a strong foundation for designing culturally tailored, gender-responsive, and cost-effective strategies to strengthen parental FL and promote healthier family nutrition practices in Egypt.

## Conclusions and recommendations

In conclusion, this study provides the first comprehensive assessment of parental FL in Egypt, revealing that while 58.6% of parents demonstrated adequate overall FL, substantial gaps persist in interactive and functional domains. Mothers consistently outperformed fathers across all FL dimensions; however, this difference was largely explained by educational and socioeconomic factors rather than gender alone. The most critical gaps were related to difficulties in selecting appropriate nutrition information, assisting others with dietary decisions, interpreting nutrition labels, and identifying reliable sources of information.

Interestingly, adequate FL was positively associated with overweight and obesity. This suggests that increased nutritional awareness does not automatically translate into healthier weight outcomes. This pattern may reflect greater health consciousness among overweight individuals or barriers that hinder the practical application of nutrition knowledge in daily life. Hence, improving FL alone may not be sufficient to prevent obesity unless accompanied by behavioural, structural, and environmental supports.

Improving parental FL therefore requires multifaceted, equity-oriented interventions that enhance practical food-handling, label-reading, and communication skills while addressing structural and time-related barriers to healthy eating. Educational programs targeting fathers and younger parents, as well as community-based and workplace initiatives, could yield sustainable improvements in household nutrition.

Integrating FL education into school curricula and primary healthcare systems will promote intergenerational learning and empower families to make informed food choices. By fostering both knowledge and supportive environments, policymakers can help translate awareness into action, thereby advancing progress toward healthier diets and reducing Egypt’s dual burden of undernutrition and obesity.

## Supplementary Information

Supplementary Material 1.

Supplementary Material 2.

## Data Availability

The datasets used and/or analyzed for the current study are available from the corresponding author on reasonable request.

## Abbreviations

AOR: Adjusted odds ratio
BMI: Body mass index
CAPMS: Central Agency for Public Mobilization and Statistics
CFL: Critical food literacy
CI: Confidence interval
EP: Egyptian pounds
FAO: Food and Agriculture Organization
FFL: Functional food literacy
FL: Food literacy
IFL: Interactive food literacy
IRB: Institutional Review Board
MENA: Middle East and North Africa
OR: Odds ratio
PC: Personal computer
SFLQ: Short Food Literacy Questionnaire
SPSS: Statistical Package for the Social Sciences
TFL: Total food literacy
UN DESA: United Nations Department of Economic and Social Affairs
WHO: World Health Organization

## References

[1] UN DESA. The Sustainable Development Goals Report 2023: Special Edition - July 2023. New York, USA: UN DESA. 2023. Available from: https://unstats.un.org/sdgs/report/2023/. Accessed 2024 August 22.

[2] UNICEF Middle East and North Africa Regional Office. Nutrition Strategic Direction 2030: MENA region. UNICEF MENA; 2024. Available from: https://www.unicef.org/mena/media/25906/file/Nutrition%20Strategic%20Direction%202030%20MENA%20Main%20Document.pdf%20.pdf. cited 08 Nov 2025.

[3] Fanzo J, Davis C, McLaren R, Choufani J. The effect of climate change across food systems: implications for nutrition outcomes. Global Food Secur. 2018;18:12–9.

[4] Food and Agriculture Organization of the United Nations (FAO). The state of food security and nutrition in the world 2023: Urbanization, agrifood systems transformation and healthy diets across the rural–urban continuum. Rome: FAO; 2023.

[5] World Health Organization (WHO). Global nutrition policy review 2023: addressing malnutrition in all its forms in an era of multiple crises. Geneva: WHO; 2023.

[6] Hoteit M, Mansour R, Mohsen H, et al. Status and correlates of food and nutrition literacy among parents-adolescents’ dyads: findings from 10 Arab countries. Front Nutr. 2023;10:1151498. 10.3389/fnut.2023.1151498. 37200945 10.3389/fnut.2023.1151498 PMC10186151

[7] Vidgen HA, Gallegos D. Food literacy: time for a new term or just another buzzword? J Home Econ Inst Aust. 2011;18(2):2–8.

[8] Salah El-Din EM, Elabd MA, Nassar MS, Metwally AM, Abdellatif GA, Rabah TM et al. The Interaction of Social, Physical and Nutritive Factors in Triggering Early Developmental Language Delay in a Sample of Egyptian Children. Open Access Maced J Med Sci. 2019;7(17):2767–2774. Available from:.10.3889/oamjms.2019.642 https://www.id-press.eu/mjms/article/view/oamjms. 10.3889/oamjms.2019.642. 31844434 10.3889/oamjms.2019.642 PMC6901873

[9] El Din EMS, Rabah TM, Metwally AM, Nassar MS, Elabd MA, Shaalan A, et al. Potential risk factors of developmental cognitive delay in the first two years of life. Open Access Maced J Med Sci. 2019;7(12):2024–30. 10.3889/oamjms.2019.566. 31406549 10.3889/oamjms.2019.566 PMC6684437

[10] Al-Otaibi HH, Basuny AM. Nutritional knowledge, attitudes, and practices of parents in Saudi Arabia. J Nutr Health Sci. 2015;2(1):1–7. 10.15744/2393-9060.2.101.

[11] Cullen T, Hatch J, Martin W, Higgins JW, Sheppard R. Food literacy: definition and framework for action. Can J Diet Pract Res. 2015;76(3):140–5. 26280794 10.3148/cjdpr-2015-010

[12] Metwally AM, El-Sonbaty M, El Etreby LA, et al. Stunting and its determinants among governmental primary school children in egypt: A school-based Cross-sectional study. Open Access Maced J Med Sci. 2020;8(B):650–7. 10.3889/oamjms.2020.4757.

[13] Metwally AM, Shaaban FA, Mahmoud WS, et al. Vulnerability and weaknesses of eating habits of overweight school children as an entry risk for COVID-19. Open Access Maced J Med Sci. 2020;8(T1):158–66. 10.3889/oamjms.2020.5049.

[14] Ronto R, Ball L, Pendergast D, Harris N. Food literacy at secondary schools in Australia. J Sch Health. 2016;86(11):823–8. 27714873 10.1111/josh.12440

[15] Saleh F, Ara F, Mumu SJ, Hafez MA. Assessment of food security in the MENA region: the case of Palestine. Public Health Nutr. 2015;18(1):123–32.

[16] Garcia IL, Fernald LCH, Aboud FE, Otieno R, Alu E, Luoto JE. Father involvement and early child development in a low-resource setting. Soc Sci Med. 2022;302:114933. 10.1016/j.socscimed.2022.114933. 35472657 10.1016/j.socscimed.2022.114933 PMC9262343

[17] Ng NBH, Ng JQX, Shen L, Shorey S. Redefining roles-Fathers play a crucial role in shaping children’s healthy eating behaviors: Cross-Sectional observational study. Nutrients. 2025;17(5):860. 10.3390/nu17050860. 40077731 10.3390/nu17050860 PMC11901718

[18] Metwally AM, Helmy MA, Salah El-Din EM, et al. National screening for Egyptian children aged 1 year up to 12 years at high risk of autism and its determinants: a step for determining what ASD surveillance needs. BMC Psychiatry. 2023;23:471. 10.1186/s12888-023-04977-5. 37381024 10.1186/s12888-023-04977-5 PMC10304233

[19] Hahn GJ, Meeker WQ. Statistical intervals. New York, NY: Wiley; 1991.

[20] El Hamidi F. Energy Subsidy Reform in Egypt: The Gender–‘Energy’Poverty Nexus. SSRN Working Paper 1055; 2016. Available from: https://ssrn.com/abstract=3054085.

[21] Assad R, Krafft C. The structure and evolution of employment in Egypt 1998–2012. Economic Res Forum (ERF) Working Paper 805. 2012. 10.1093/acprof:oso/9780198737254.003.0002. Egypt.

[22] World Health Organization. WHO housing and health guidelines. Geneva, Switzerland: World Health Organization; 2018.

[23] Historical census of housing tables: crowding. Washington, DC: U.S. Census Bureau, Housing and Household Economic Statistics Division; 2011.

[24] Gréa Krause C, Beer-Borst S, Sommerhalder K, Hayoz S, Abel T. A short food literacy questionnaire (SFLQ) for adults: findings from a Swiss validation study. Appetite. 2018;120:275–80. 10.1016/j.appet.2017.08.039. 28912107 10.1016/j.appet.2017.08.039

[25] Koca B, Arkan G. The relationship between adolescents’ nutrition literacy and food habits, and affecting factors. Public Health Nutr. 2020;24(4):717–28. 10.1017/S1368980020001494. 10.1017/S1368980020001494 PMC11574834 32723409

[26] World Health Organization. AnthroPlus for personal Computers. Manual software for assessing growth of the world’s children and adolescents. Geneva, Switzerland: World Health Organization; 2009.

[27] World Health Organization. Physical Status: The Use and Interpretation of Anthropometry. Report of a WHO Expert Committee. Technical Report Series No. 854. Geneva, Switzerland: World Health Organization. 1995. 10.1037/e412352004-001. 8594834

[28] IBM Corp. IBM SPSS statistics for Windows, version 26.0. Armonk, NY: IBM Corp; 2019.

[29] Metwally AM, Elmosalami DM, Elhariri H, et al. Accelerating hepatitis C virus elimination in Egypt by 2030: A National survey of communication for behavioral development as a modelling study. PLoS ONE. 2021;16(2):e0242257. 33621232 10.1371/journal.pone.0242257 PMC7901784

[30] Metwally AM, El-Sonbaty M, Elmosalami D, et al. Assessing the effective communication channels to reduce child and adolescent marriage in rural communities of Egypt. Open Access Maced J Med Sci. 2021;9:1288–99. 10.3889/oamjms.2020.4766.

[31] Metwally AM, Basha WA, Elshaarawy GA, et al. How did the use of the social marketing approach in Egyptian communities succeed in improving breastfeeding practices and infants’ growth? BMC Public Health. 2024;24:1298. 10.1186/s12889-024-18469-y. 38741049 10.1186/s12889-024-18469-y PMC11089676

[32] Metwally AM, Sallam SF, Mawla MAA, et al. Promoting weaning practices and growth of Egyptian infants by using communication for behavioral development approach. BMC Pediatr. 2022;22:689. 10.1186/s12887-022-03741-0. 36456920 10.1186/s12887-022-03741-0 PMC9713754

[33] Metwally AM, Hanna C, Galal YS, et al. Impact of nutritional health education on knowledge and practices of mothers of anemic children in El Othmanyia Village – Egypt. Open Access Maced J Med Sci. 2020;8(E):458–65. 10.3889/oamjms.2020.4766.

[34] Metwally AM, Soliman M, Abdelmohsen AM, et al. Effect of counteracting lifestyle barriers through health education in Egyptian type 2 diabetic patients. Open Access Maced J Med Sci. 2019;7(17):2886–94. 10.3889/oamjms.2019.624. 31844454 10.3889/oamjms.2019.624 PMC6901843

[35] Salah EM, Khalifa AG, Metwally AM, Abdel Hamid N, Hussien HA, Moneer ZM. The impact of school snacks on cognitive function of primary school children in Egypt. J Appl Sci Res. 2012;8(12):5639-650.

[36] Metwally AM, El-Sonbaty MM, El Etreby LA, Salah El-Din EM, Abdel Hamid N, Hussien HA, Hassanin AM, Monir ZM. Impact of National Egyptian school feeding program on growth, development, and school achievement of school children. World J Pediatr. 2020;16(4):393–400. 10.1007/s12519-020-00342-8. 32056148 10.1007/s12519-020-00342-8

[37] Silva P. Food and nutrition literacy: exploring the divide between research and practice. Foods. 2023;12(14):2751. 10.3390/foods12142751. 37509843 10.3390/foods12142751 PMC10378922

[38] Johnson L, Williams R, Thompson P. Nutrition and food literacy: framing the challenges to health communication. Public Health Nutr. 2023;26(11):1895–903. 10.3390/nu15224708 PMC10674981 38004102

[39] Ostarelli V, Michou M, Panagiotakos DB, Lionis C. Parental health literacy and nutrition literacy affect child feeding practices: A cross-sectional study. Nutr Health. 2022;28(1):59–68. 10.1177/02601060211001489. 33913343 10.1177/02601060211001489

[40] Silva B, Lima JPM, Baltazar AL, Pinto E, Fialho S. Perception of Portuguese consumers regarding food labeling. Nutrients. 2022;14:2944. 35889901 10.3390/nu14142944 PMC9323138

[41] Hoteit M, Mohsen H, Hanna-Wakim L, Sacre Y. Parent’s food literacy and adolescents nutrition literacy influence household’s food security and adolescent’s malnutrition and anemia: findings from a National representative cross sectional study. Front Nutr. 2022;9:1053552. 10.3389/fnut.2022.1053552. 36643976 10.3389/fnut.2022.1053552 PMC9837989

[42] Nayga RM, Lipinski D, Savur N. Consumers’ use of nutritional labels while food shopping and at home. J Consum Aff. 1998;32:106–20.

[43] Davis K, Miller S, Anderson J. A scoping review of food and nutrition literacy programs. Nutr Rev. 2023;81(4):345–59.

[44] Bhawra J, Kirkpatrick SI, Hall MG, Vanderlee L, Thrasher JF, Hammond D. Correlates of self-reported and functional Understanding of nutrition labels across 5 countries in the 2018 international food policy study. J Nutr. 2022;152:S13–24. 10.1093/jn/nxac018 PMC9188861 35274701

[45] Smith A, Jones B, Brown C. Gender differences in food literacy: A review of the literature. J Nutr Educ Behav. 2020;52(4):345–52. 10.1016/j.jneb.2019.12.005.

[46] Krause C, Sommerhalder K, Beer-Borst S. Nutrition-specific health literacy: development and testing of a multi-dimensional questionnaire. Ernahrungs Umschau. 2018;65(4):80–9.

[47] Palumbo R, Annarumma C, Adinolfi P, et al. Crafting and applying a tool to assess food literacy: findings from a pilot study. Trends Food Sci Technol. 2017;67:173–82.

[48] Nayga RM. Nutrition knowledge, gender, and food label use. J Consum Aff. 2000;34:97–112.

[49] Caraher M, Baker H, Burns M. Children’s views of cooking and food Preparation. Br Food J. 2004;106(4):255–73.

[50] Al-Hazzaa HM, Abahussain NA, Al-Sobayel HI, Qahwaji DM, Musaiger AO. Physical activity, sedentary behaviors and dietary habits among Saudi adolescents relative to age, gender and region. Int J Behav Nutr Phys Act. 2011;8(1):140. 10.1186/1479-5868-8-140. 22188825 10.1186/1479-5868-8-140 PMC3339333

[51] Bailey RL, Gahche JJ, Lentino CV, et al. Dietary supplement use in the united States, 2003–2006. J Nutr. 2011;141(2):261–6. 21178089 10.3945/jn.110.133025 PMC3021445

[52] Begley A, Paynter E, Butcher LM, Dhaliwal SS. Examining the association between food literacy and food insecurity. Nutrients. 2019;11(2):445. 30791670 10.3390/nu11020445 PMC6412525

[53] Thomas H, Azevedo Perry E, Slack J, Samra HR, Manowiec E, et al. Complexities in conceptualizing and measuring food literacy. J Acad Nutr Diet. 2019;119(4):563–73. 10.1016/j.jand.2018.10.015. 30670348 10.1016/j.jand.2018.10.015

[54] Elemile MG, Bello CB, Ajayi K, Akinwale OD. Health literacy and effects on household/family dietary behaviour: a systematic scoping review. J Public Health. 2023;31:1–15.

[55] Tabacchi G, Battaglia G, Alesi M, et al. Food literacy predictors and associations with physical and emergent literacy in pre-schoolers: results from the Training-to-Health project. Public Health Nutr. 2020;23(2):356–65. 31474231 10.1017/S1368980019002404 PMC10201352

[56] Devine CM, Connors M, Bisogni CA, Sobal J. Life-course influences on fruit and vegetable trajectories: qualitative analysis of food choices. J Nutr Educ. 1998;30(6):361–70.

[57] Truman E, Lane D, Elliott C. Nutrition and food literacy: framing the global perspective. Nutrients. 2023;15(22):4708. 38004102 10.3390/nu15224708 PMC10674981

[58] Manna A, Vidgen H, Gallegos D. Examining the effectiveness of food literacy interventions in improving food literacy behavior and healthy eating among adults belonging to different socioeconomic groups: a systematic scoping review. Syst Rev. 2024;13:221. 39198926 10.1186/s13643-024-02632-y PMC11350956

[59] Doustmohammadian A, Omidvar N, Keshavarz-Mohammadi N, et al. The association and mediation role of food and nutrition literacy (FNLIT) with eating behaviors, academic achievement and overweight in 10–12 years old students: a structural equation modeling. Nutr J. 2022;21:45. 35778743 10.1186/s12937-022-00796-8 PMC9248125

[60] Wang Y, Hu C, Yang X, Zhang J. Evaluation of the nutrition literacy assessment questionnaire for college students and identification of the influencing factors of their nutrition literacy. BMC Public Health. 2023;23:2127. 37904128 10.1186/s12889-023-17062-z PMC10617111

[61] Vaitkeviciute R, Ball LE, Harris N. The relationship between food literacy and dietary intake in adolescents: a systematic review. Public Health Nutr. 2023;26(5):789–801. 10.1017/S1368980014000962 PMC10271388 24844778

[62] Dallacker M, Hertwig R, Mata J. The frequency of family meals and nutritional health in children: a meta-analysis. Obes Rev. 2018;19(5):638–53. 10.1111/obr.12659. 29334693 10.1111/obr.12659

[63] Worsley A, Wang WC, Byrne S, Yeatman H. Different patterns of Australian adults’ knowledge of foods and nutrients related to metabolic disease risk. J Nutr Sci. 2014;3:e14. 10.1017/jns.2014.12. 25191606 10.1017/jns.2014.12 PMC4153087

[64] Worsley A, Wang WC, Byrne S, Yeatman H. Patterns of food literacy among Australian parents. Appetite. 2021;161:105125.33482302

[65] Velardo S. The nuances of health literacy, nutrition literacy, and food literacy. J Nutr Educ Behav. 2015;47(4):385–9. 26026651 10.1016/j.jneb.2015.04.328

[66] Pelletier JE, Laska MN. Balancing healthy meals and busy lives: associations between work, school, and family responsibilities and perceived time constraints among young adults. J Nutr Educ Behav. 2012;44(6):481–9. 10.1016/j.jneb.2012.04.001. 23017891 10.1016/j.jneb.2012.04.001 PMC3496024

[67] Bailey RL, Gahche JJ, Miller PE, Thomas PR, Dwyer JT. Why US adults use dietary supplements. JAMA Intern Med. 2013;173(5):355–61. 10.1001/jamainternmed.2013.2299. 23381623 10.1001/jamainternmed.2013.2299

[68] Slater J, Falkenberg T. Assessing food and nutrition literacy in children and adolescents: a systematic review of existing tools. Public Health Nutr. 2023;26(3):456–68. 10.1017/S1368980021004389 PMC9991546 34728004

[69] Kamel IH, Metwally AM, Zaki DA, et al. Nutrition literacy across adolescence stages in egypt: a quartile-based analysis for tailored educational strategies. BMC Public Health. 2025;25:2389. 10.1186/s12889-025-23583-6. 40618101 10.1186/s12889-025-23583-6 PMC12228376

[70] Nasreddine L, Ayoub JJ, Al Jawaldeh A. Review of the nutrition situation in the Eastern mediterranean region. East Mediterr Health J. 2018;24(1):77–91. 29658624

[71] World Health Organization (WHO). Promoting healthy diets. WHO Western Pacific Region; c2023. Available from: https://www.who.int/westernpacific/activities/promoting-healthy-diets. cited 08 Nov 2025.

[72] Azevedo Perry E, Thomas H, Samra HR, Edmonstone S, Davidson L, Faulkner A, Petermann L, Manafò E, Kirkpatrick SI. Identifying attributes of food literacy: a scoping review. Public Health Nutr. 2017;20(13):2406–15. 10.1017/S1368980017001276. 28653598 10.1017/S1368980017001276 PMC10261432

[73] Vidgen HA, Gallegos D. Development of food literacy in children and adolescents: implications for the design of strategies to promote healthier and more sustainable diets. Nutr Rev. 2023;82(4):536–48. 10.1093/nutrit/nuad072 PMC10925906 37339527

[74] Savage JS, Fisher JO, Birch LL. Parental influence on eating behavior: conception to adolescence. J Law Med Ethics. 2007;35(1):22–34. 10.1111/j.1748-720X.2007.00111.x. 17341215 10.1111/j.1748-720X.2007.00111.x PMC2531152

[75] Cabezas MF, Nazar G. A scoping review of food and nutrition literacy programs. Health Promot Int. 2023;38(5):daad090. 10.1093/heapro/daad090. 37676303 10.1093/heapro/daad090

[76] Schaafsma HN, Caruso OT, McEachern LW, Seabrook JA, Gilliland JA. Understanding food literacy intervention effectiveness: postsecondary students’ perspectives on how a mHealth food literacy intervention impacted their dietary behaviors. J Nutr Educ Behav. 2025;57(4):304–15. 10.1016/j.jneb.2025.01.003. 39891647 10.1016/j.jneb.2025.01.003

[77] Council for International Organizations of Medical Sciences (CIOMS). International ethical guidelines for biomedical research involving human subjects. Geneva: CIOMS; 2016. 40523065

[78] Metwally AM, Amer HA, Salama HI, Abd El Hady SI, Alam RR, Aboulghate A, et al. Egyptian patients’/guardians’ experiences and perception about clinical informed consent and its purpose: cross sectional study. PLoS ONE. 2021;16(6):e0252996. 34125842 10.1371/journal.pone.0252996 PMC8202917

