# Supplementary material for: Status and predictors of parental food literacy: an Egyptian insight to highlight gaps and challenges
Source: BMC Public Health. 2026 Feb 2;26:729. doi: 10.1186/s12889-026-26249-z (PMC12931042; doi:10.1186/s12889-026-26249-z)
Supplement: Supplementary file 1 — Supplementary Material 1. [file 12889_2026_26249_MOESM1_ESM.docx]

| serial | **Gov.** | **classification according to HDI** | **Hai (Kesm/Markaz)** | **Urban** | | | **Rural** | | | | Total | |
| --- | --- | --- | --- | --- | --- | --- | --- | --- | --- | --- | --- | --- |
|  |  |  |  | **Shiakha**  **(HH)** | **Urban target n** | **k (Urban)** | **Local Unit** | **Village** | **HH** | **children** | **Total HH** | **Total target n** |
| 1 | Cairo | **High** | **Al Nozhah** | **Al Hicksit (1390)** | **50** | **28** |  |  |  |  | **1390** | **50** |
|  |  | **middle** | **Al Saiedah Zainab** | **Alkabsh (1390)** | **50** | **28** |  |  |  |  | **1390** | **50** |
|  |  | **low** | **el Sharabia** | **Al Amiria (1390)** | **50** | **28** |  |  |  |  | **1390** | **50** |
| 2 | Dakhleya | **High** | **Al Senbelawin** | **Al Sinblaween city (317)** | **26** | **12** | **Kafr Alruwk** | **Alshalaa**  **(905)** | **74** | **12** | **1222** | **100** |
|  |  | **middle** | **Markaz of MietSalsil** | **Mit salsil city (317)** | **26** | **12** | **Alatihad** | **AlJafara (905)** | **74** | **12** | **1222** | **100** |
|  |  | **low** | **Al Gamaliah** | **Almataria city (317)** | **26** | **12** | **Alsafra** | **Al Dahear**  **(905)** | **74** | **12** | **1222** | **100** |
| 4 | Fayoum | **High** | **Al Fayoum City** | **Alqism rabie (145)** | **20** | **7** | **Dacia** | **Al Sunbat (580)** | **80** | **7** | **725** | **100** |
|  |  | **middle** | **Markazof Senoures** | **Senoures (145)** | **20** | **7** | **Terrsa** | **Alzawia El Khadra (580)** | **80** | **7** | **725** | **100** |
|  |  | **low** | **Markazof Tamiaha** | **Tamiaha (145)** | **20** | **7** | **Sarsna** | **Kafr Omira (580)** | **80** | **7** | **725** | **100** |
| 8 | MarsaMatrouh | **High** | **MarsaMatrouh** | **MarsaMatrouh**  **(Alsanusia & Kilo 4) (470)** | **68** | **7** | **Alkasr** | **Alkasr**  **(220)** | **32** | **7** | **690** | **100** |
|  |  | **middle** | **Al Hamam City** | **Al Hamam City (470)** | **68** | **7** | **Alsalam** | **Alsalam (220)** | **32** | **7** | **690** | **100** |
|  |  | **low** | **KismSaiedy Barany** | **AlNajyla (470)** | **68** | **7** | **Almathany** | **Almathany (220)** | **32** | **7** | **690** | **100** |
| total | | | | **6966** | **492** |  | **5115** | | **558** |  | **12081** | **1,050** |

**S- table 1: Targeted households (HH), target enrolled children (n), and sampling interval (k) by governorate, socioeconomic stratum, and locality**

Notes: HH indicates the total number of eligible households in the locality household list. Target enrolled children (n) were allocated within each governorate and socioeconomic stratum according to the study sampling plan (Cairo: 50 per stratum; other governorates: 100 per stratum) and distributed between urban and rural localities proportionally to HH. The sampling interval k was computed as k = HH / n (rounded to the nearest integer) for systematic random sampling (every kth household).
