# Supplementary material for: Status and predictors of parental food literacy: an Egyptian insight to highlight gaps and challenges
Source: BMC Public Health. 2026 Feb 2;26:729. doi: 10.1186/s12889-026-26249-z (PMC12931042; doi:10.1186/s12889-026-26249-z)
Supplement: Supplementary file 2 — Supplementary Material 2. [file 12889_2026_26249_MOESM2_ESM.docx]

# STROBE Checklist (Cross-sectional Study)

Manuscript: Parental Food Literacy Status and Level (Egypt). This checklist is completed in alignment with the STROBE Statement for cross-sectional studies.

| **Item** | **STROBE Recommendation (Cross-sectional)** | **Addressed in Manuscript (Section / Location)** | **Notes / Actions (if any)** |
| --- | --- | --- | --- |
| 1a | Indicate the study’s design with a commonly used term in the title or the abstract. | Title; Abstract (first sentence). |  |
| 1b | Provide an informative and balanced summary of what was done and what was found. | Abstract (Background/Methods/Results/ Conclusions). |  |
| 2 | Explain the scientific background and rationale for the investigation being reported. | Introduction (Background and rationale). |  |
| 3 | State specific objectives, including any prespecified hypotheses. | Introduction (Study aim/objectives). |  |
| 4 | Present key elements of study design early in the paper. | Methods: Study design and participants. |  |
| 5 | Describe the setting, locations, and relevant dates, including periods of recruitment, exposure, follow-up, and data collection. | Methods: Setting/location; Study period (Jan–Dec 2022). |  |
| 6a | Give eligibility criteria, and the sources and methods of selection of participants. | Methods: Inclusion/exclusion; Sampling strategy; Household selection. |  |
| 6b | For matched studies, give matching criteria and number of exposed and unexposed. (Not applicable for cross-sectional, unless matching used.) | Not applicable (no matching). | N/A. |
| 7 | Clearly define all outcomes, exposures, predictors, potential confounders, and effect modifiers. Give diagnostic criteria, if applicable. | Methods: Variables/Measures (TFL and subscales; socio-demographic; BMI; chronic disease; supplements). |  |
| 8 | For each variable of interest, give sources of data and details of methods of assessment (measurement). Describe comparability if >1 group. | Methods: Study tools and data collection; Instrument description; BMI and supplement definitions. |  |
| 9 | Describe any efforts to address potential sources of bias. | Methods: Sampling strategy; Interviewer training; Discussion: Strengths & limitations (bias). |  |
| 10 | Explain how the study size was arrived at. | Methods: Sample size calculation and targeted/completed sample. |  |
| 11 | Explain how quantitative variables were handled in the analyses; if applicable, describe which groupings were chosen and why. | Methods: BMI categorization; FL scoring; cut-offs. |  |
| 12a | Describe all statistical methods, including those used to control for confounding. | Methods: Statistical analysis (bivariate tests; multivariable logistic regression; covariate selection). |  |
| 12b | Describe any methods used to examine subgroups and interactions. | Not applicable (no planned subgroup/interaction analyses reported). | N/A. |
| 12c | Explain how missing data were addressed. | Methods: Statistical analysis (missing-data handling). |  |
| 12d | If applicable, describe analytical methods taking account of sampling strategy. | Methods: Multistage stratified random sampling described. |  |
| 12e | Describe any sensitivity analyses. | Not applicable (no sensitivity analyses reported). | N/A. |
| 13a | Report numbers of individuals at each stage of study (e.g., eligible, included, analyzed). | Methods: participation rate and declining rate; Results: participants included in analysis. |  |
| 13b | Give reasons for non-participation at each stage. | Methods: declining rate reported in “Sample size and type” |  |
| 13c | Consider use of a flow diagram. | Optional (We have provided Details of the selected governorates, localities, and socioeconomic classifications are presented in Supplementary Table S1 (Targeted Households for Parents). |  |
| 14a | Give characteristics of study participants and information on exposures and potential confounders. | Results: Descriptive characteristics (Table 1). |  |
| 14b | Indicate number of participants with missing data for each variable of interest. | Methods/Results: as the first sentence of Results. |  |
| 15 | Report numbers of outcome events or summary measures. | Results: TFL adequacy proportion; subscale results; Tables/Figures. |  |
| 16a | Give unadjusted estimates and, if applicable, confounder-adjusted estimates and their precision (e.g., 95% CI). Make clear which confounders were adjusted for and why included. | Results: Bivariate ORs (Tables 3–4) and adjusted ORs (Table 5); Methods: covariate selection. |  |
| 16b | Report category boundaries when continuous variables were categorized. | Methods: BMI and FL scoring cut-offs; Tables. |  |
| 17 | Report other analyses done—e.g., analyses of subgroups and interactions, and sensitivity analyses. | Not applicable (no additional analyses). | N/A. |
| 18 | Summarize key results with reference to study objectives. | Discussion/Conclusions. |  |
| 19 | Discuss limitations of the study, taking into account sources of potential bias or imprecision. Discuss both direction and magnitude of any potential bias. | Discussion: Strengths and limitations. |  |
| 20 | Give a cautious overall interpretation of results considering objectives, limitations, multiplicity of analyses, results from similar studies, and other relevant evidence. | Discussion. |  |
| 21 | Discuss the generalisability (external validity) of the study results. | Discussion: Strengths and limitations; Conclusions. |  |
| 22 | Give the source of funding and the role of the funders for the present study and, if applicable, for the original study on which the present article is based. | Declarations: Funding; Competing interests. |  |
